# Contribution of urinary tract infection to the burden of febrile illnesses in young children in rural Kenya

**DOI:** 10.1371/journal.pone.0174199

**Published:** 2017-03-21

**Authors:** Wechuli Geoffrey Masika, Wendy Prudhomme O’Meara, Thomas L. Holland, Janice Armstrong

**Affiliations:** 1 Department of Family Medicine, Webuye Sub-County Hospital, Webuye, Kenya; 2 Department of Family Medicine, Kabarak University, Kabarak, Kenya; 3 Department of Medicine, Duke University School of Medicine, Durham, North Carolina, United States of America; 4 Duke Global Health Institute, Durham, North Carolina, United States of America; 5 School of Public Health, Moi University College of Health Sciences, Eldoret, Kenya; Universidade Nova de Lisboa Instituto de Higiene e Medicina Tropical, PORTUGAL

## Abstract

**Introduction:**

The clinical features of UTI in young children may not localize to the urinary tract and closely resemble other febrile illnesses. In malaria endemic areas, a child presenting with fever is often treated presumptively for malaria without investigation for UTI. Delayed or inadequate treatment of UTI increases the risk of bacteremia and renal scarring in young children and subsequently complications as hypertension and end stage renal disease in adulthood.

**Methods:**

A cross-sectional study was carried out in a hospital in western Kenya. Inpatients and outpatients 2 months to five years with axillary temperature ≥37.5°C and no antibiotic use in the previous week were enrolled between September 2012 and April 2013. Urine dipstick tests, microscopy, and cultures were done and susceptibility patterns to commonly prescribed antibiotics established. UTI was defined as presence of pyuria (a positive urine dipstick or microscopy test) plus a positive urine culture.

**Results:**

A total of 260 subjects were recruited; 45.8% were female and the median age was 25months (IQR: 13, 43.5). The overall prevalence of UTI was 11.9%. Inpatients had a higher prevalence compared to outpatients (17.9% v 7.8%, p = 0.027). UTI co-existed with malaria but the association was not significant (OR 0.80, p = 0.570). The most common organisms isolated were *Escherichia coli* (64.5%) and *Staphylococcus aureus* (12.9%) and were sensitive to ciproflaxin, cefuroxime, ceftriaxone, gentamycin and nitrofurantoin but largely resistant to more commonly used antibiotics such as ampicillin (0%), amoxicillin (16.7%), cotrimoxazole (16.7%) and amoxicillin-clavulinate (25%).

**Conclusion:**

Our study demonstrates UTI contributes significantly to the burden of febrile illness in young children and often co-exists with other infections. Multi-drug resistant organisms are common therefore choice of antimicrobial therapy should be based on local sensitivity pattern.

## Introduction

Acute febrile illnesses are common in children worldwide, especially in those under 5-years of age. Infections leading to these febrile episodes are responsible for the majority of under-5 mortality [1]. Febrile illness is typically presumptively treated as malaria in African children in endemic areas, where the majority of these children are given anti-malarial drugs [2–4]. World Health Organization guidelines have been developed to guide empiric treatment at first-level facilities [5, 6] with an emphasis on anti-malarial administration. Yet presence of fever alone is not adequate to distinguish malaria from other infectious conditions, especially if the presentation is without focal signs. It is estimated that more than half of children presenting to public health facilities in Africa do not have malaria infection [7] thus presumptive treatment of fever is not justifiable [8]. Presumptive treatment for malaria in children who do not have malaria could lead to delays in treating the real underlying condition and escalate development of anti-malarial drug resistance. Evidence is emerging to describe the etiologies of non-malaria febrile illness [9–12] particularly in areas where the burden of malaria is declining. However, these studies focus mainly on vector borne diseases and other diseases of the tropics.

Urinary tract infections (UTI) in infancy and childhood can present with clinical features that may not localize to the urinary tract and may closely resemble other febrile illnesses without focal signs. Furthermore, UTI may co-exist with these common childhood illness [13]. Where clinical diagnosis alone is employed in management of pediatric fevers, UTI may be missed or its treatment delayed. Such children are at risk of developing bacteremia and more serious illness. Long-term prospective studies suggest a link between childhood renal scarring arising from untreated UTI and possible long-term complications in adulthood such as hypertension and end stage renal disease [14].

Data from a rural district hospital in western Kenya demonstrate that UTI diagnosis is uncommon. In 2011, 2% of cases had clinically diagnosed UTI but not as a primary diagnosis. Without active investigation for UTI such data may not reflect the real burden of UTI, especially in children under 5 years. This study was conducted in a malaria endemic region in Western Kenya among febrile children aged between 2 months to 5 years to determine the burden of UTI, causative bacterial pathogens and their antimicrobial sensitivity pattern.

## Materials and methods

This study was conducted at Webuye Sub-county Hospital, a government hospital in Kenya located in Western Province, Bungoma County along the Nairobi-Uganda highway, approximately 400km northwest of Nairobi. The hospital serves as a referral centre for lower-level health facilities in the Sub-county. In a typical year, the hospital admits approximately 12500 patients, including 3200 pediatric patients. Of this, 2600 are under 5 year old children. The pediatric ward has 56 beds and admits on average 250 children per month. Most of the patients are referred from or come as self-referrals after treatment has been initiated at lower-level facilities.

### Study population

The study population consisted of children aged between 2 months and 5 years presenting to Webuye Sub-county Hospital’s pediatric department during the study period. Children presenting to the under-5 outpatient department are triaged to the pediatric ward or the pediatric stabilization center if they cannot be managed as outpatients. Children identified at any of these departments were eligible. To achieve our study objective a minimum sample size of 173 children was calculated using Fisher’s formula using a prevalence of 13.3% from a study in Nairobi in 2004 [15]. We surpassed this to recruit 260 children over the study period. In order to spread enrollment across a longer time period, every second child with fever was sampled using a simple alternate consecutive sampling method until the required sample size was achieved.

An age range of 2 months to 5 years was chosen because of higher risk of UTI and renal scarring [11, 16]. The criterion for consideration for inclusion into the study was axillary temperature ≥37.5°C. Axillary temperature is generally lower than core temperature, thus a cutoff of 37.5°C was used. Neonates were excluded for two reasons: 1) their clinical presentations are unique, e.g. with hypothermia instead of fever, and 2) the need for immediate empiric antibiotics for the febrile neonate—in accordance with management guidelines of neonatal sepsis—which would confound interpretation of subsequent urine studies. The study also excluded children with known urologic conditions on management or follow up and those who had used antibiotics within a week prior to presentation.

### Sample collection

Following cleaning of the perineum with normal saline cotton swabs, children older than two years who were able to follow instructions from their parents provided a mid-stream or clean catch sample of urine directly into two sterile urine bottles under observation by one of the research staff. Where two adequate samples could not be obtained, the collected sample was divided into two bottles. Sterile feeding tube size 5 or 6 was used for in-and out catheterization on children younger than 2 years or those not able to follow instructions. The first sample was utilized for dipstick and microscopy and the second sample for urine culture.

### Laboratory procedures

All children enrolled in the study were initially screened using both urine dipstick test (nitrite and leukocyte esterase) and urine microscopy [17, 18]. Dipstick test (URISCAN, YD diagnostics CORP. 76, Seori-ro, Republic of Korea) was carried out on the first sample and results read within 60 seconds for presence or absence of leukocyte esterase (LE) and nitrites (N). The dipstick sample was used for microscopy testing which was done within an hour of sample collection. Samples were centrifuged, supernatant discarded, and the sediment used to prepare a smear on a slide. This was examined following established standard operating procedures under microscope at high power (x1000). Results were recorded as Negative, or + (if <5WBC/hpf), ++ (5-9WBC/hpf) or +++ (>10WBC/hpf) in the data collection tool. A positive test on microscopy was considered as ≥5WBC/hpf.

If the result on the first sample was positive on microscopy and/or dipstick, the second sample was then submitted for culture and subsequent antimicrobial sensitivity testing. If both dipstick and microscopy were negative, culture of the second sample was not performed, as bacteriuria in the absence of pyuria, leukocyte esterase, or nitrite is unlikely to represent true UTI. Samples for culture were inoculated on Cysteine lactose electrolyte deficient (CLED) media and incubated for 24–48 hours. Significant growth was considered as growth of pure colonies of organisms that are non-contaminants and meeting a threshold of ≥50,000 colony forming units (CFU)/ml [16]. Results of growth on culture plates, and species identification with Gram stain slides, Triple Sugar Iron (TSI), oxidase, catalase and coagulase tests were captured on camera and confirmed by a second technologist. The laboratory technologists carrying out urine microscopy and culture were blind to one another’s results.

Antimicrobial sensitivity testing was carried out by Kirby Bauer disc diffusion technique [19, 20], according to the recommendations of the Clinical and Laboratory Standards Institute (CLSI) [21] using commercially available discs on Mueller Hinton Agar Plates. The isolates were tested against common antimicrobials used at first-level facilities: trimethoprim-sulfamethoxazole (TMP-SMX), amoxicillin, nitrofurantoin, amoxicillin-clavulanate, nalidixic acid, ampicillin, gentamicin, cefuroxime, ceftriaxone and ciprofloxacin.

The definition of UTI in our study was taken as a positive test result for pyuria by either microscopy or dipstick test and a positive growth on culture of at least 50000 CFU of a single uropathogen [16].

### Data handling and analysis

Patient information, including socio-demographic details and history, was obtained upon enrollment and the children examined for fever (axillary temperature) with a digital thermometer. Data extracted from patient files included the clinician’s impression of the child’s illness or assigned clinical diagnoses, results of malaria microscopy, antibiotics prescribed and discharge diagnoses after management.

Data were entered into an Access database and exported to STATA v.11 for analysis. In addition to the basic descriptive analyses, subjects were stratified according to gender, age group, mode of admission, circumcision status and primary diagnosis assigned by clinician. Prevalence of UTI among the various strata and the odds of UTI for discharge diagnosis of malaria were calculated using Chi- square.

### Ethical considerations

Approval to carry out research was obtained from the Institutional Research and Ethics Committee (IREC) of Moi University School of Medicine and the Medical superintendent of Webuye Sub-county Hospital. Written informed consent was obtained from parents or caretakers of the children who qualified for inclusion in the study.

All children were treated for their admission diagnoses using established hospital protocols and the current basic pediatric protocols of Kenya [22]. Outpatients with suspected UTI on dipstick test were given empiric treatment with amoxicillin-clavulanate followed by a mobile phone call to the guardian to convey culture results and whether there was need to change antibiotics based on sensitivity results. Those admitted to the ward with suspected UTI received a 1–2 day course of parenteral gentamicin or ceftriaxone if this was not already included under the patient’s treatment plan for the admission diagnoses; followed by an appropriate oral antibiotic depending on sensitivity results to complete a 7 day treatment course.

## Results

**Fig 1:**
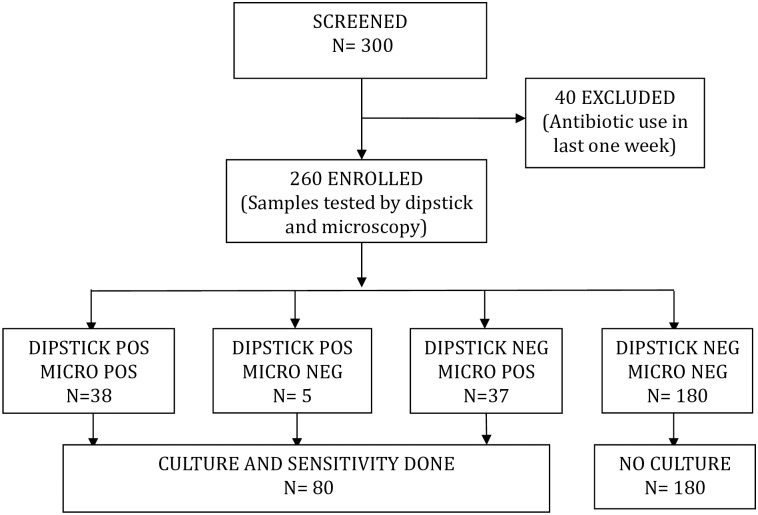
Study enrollment and urine dipstick test results diagram.

Patients were enrolled between September 2012 and April 2013. A total of 300 febrile children were screened; forty (13.3%) children were excluded because of prior use of antibiotics (Fig 1).

Of the remaining 260 children, 80(30.8%) had pyuria determined by positive urine dipstick testing and/or significant pyuria on microscopy. These 80 samples were submitted for culture.

### Demographic characteristics

A total of 106 (40.8%) children were inpatients and 154 (59.2%) were outpatients. One hundred and nineteen (45.8%) were female while 141 (54.2%) were male. The median age in months of subjects in this study was 25 (IQR) (13, 43.5). Only one child was circumcised (Table 1).

**Table 1:** Demographic characteristics of febrile under-5 children.

VARIABLE	FREQUENCY (%)N = 260
**Age (Months)**	
2 months- 24 months	129(49.6%)
25 months –59 months	131(50.4%)
**Gender**	
Female	119(45.6%)
Male	141(54.4%)
**Urine sample method collection**	
Catheterization	127(48.8%)
Clean catch/midstream	133(51.2%)

Urine samples from 127 (48.8%) children were obtained through a sterile in-and—out catheterization, while the rest gave either a midstream urine sample or a clean catch sample.

#### Correlation of body temperature and UTI in children

Fever (axillary temperature) of 37.5°C was used as the entry point into this study. Of the Children who had UTI, 23 (74.2%) had a temperature of 38°C and above (Table 2). However, only eight children presented with a temperature above 39°C.

**Table 2:** Correlation of urinary tract infection with body temperature.

TEMP RANGE (°C)	FREQUENCY (%)	UTI
37.5–37.9	77 (29.6)	8
38.0–38.4	69 (26.5)	7
38.5–38.9	47 (18.1)	8
39.0–39.4	43 (16.5)	5
39.5–39.9	13 (5.0)	2
≥ 40.0	11 (4.3)	1
	**TOTAL**	**31**

### Results of testing

Forty-three children tested positive for either LE or N or both (positive dipstick), 38 of whom also had pyuria by microscopy. Five children had a positive dipstick but negative urine microscopy and 37 children had pyuria by microscopy with a negative dipstick (Fig 1). Samples from all children with a positive dipstick or pyuria by microscopy were cultured.

Thirty-one children had both pyuria and significant growth of a single bacterial uropathogen thus fulfilling the criteria for UTI, while four samples grew contaminants. Of the tests that had both dipstick and microscopy positive, 73.7% (28/38) had a positive urine culture while only 5.4% (2/37) of the tests with microscopy positive and dipstick negative had a positive culture. Twenty percent (1/5) of the tests that had dipstick positive and microscopy negative had a positive culture.

#### Prevalence of urinary tract infection

The overall prevalence of UTI (defined by a positive urine culture with either a positive dipstick or pyuria by microscopy) among febrile patients in our study sample was 11.9% (31/260, 95%CI: 7.8%-16.0%) (Table 3).

**Table 3:** Prevalence of urinary tract infection by various strata.

VARIABLE	N	UTI, n (%)	P-Value
**Age (Months)**			
2 Months– 24 months	129	13 (10.1)	
25 months– 59 months	131	18 (13.8)	0.98
**Gender**			
Female	119	17 (14.3)	
Male	141	14 (9.9)	0.373
**Category of Patient**			
Inpatient	106	19 (17.9)	
Outpatient	154	12 (7.8)	0.027

There was no significant difference in the prevalence of UTI across age groups and gender. The prevalence in children admitted to the hospital was higher than that of children enrolled from the outpatient department (19(17.9%) versus 12(7.8%), p = 0.027).

#### Clinical diagnoses assigned to febrile children

Malaria was the most common clinical diagnosis assigned to patients in the study (243 of 260 [93.5%]), however only 113 (46.5%) had a positive malaria smear [Table 4]. Other clinical diagnoses included pneumonia (51, 19.6%), acute gastroenteritis (23, 8.8%), and upper respiratory tract infections (21, 8%).

**Table 4:** Co-existence of UTI with other clinical diagnoses.

	NUMBER	UTI	UTI CO-MORBIDITY
Malaria, (smear +ve)	113	12	10.6%
Clinical malaria, (smear—ve)	130	14	10.8%
Acute gastroenteritis	23	1	4.3%
Severe Acute Malnutrition	6	1	16.7%
Acute otitis media	21	1	4.8%
UTI	6	1	--
Pneumonia	51	0	0
Sickle cell disease	11	0	0
Others	32	1	3.1%

Urinary tract infection occurred in 12/113(10.6%) patients with a positive malaria smear and in 19/147(12.9%) of those who did not have malaria. The odds ratio of having a UTI in patients with parasitological-confirmed malaria was not significantly higher than in patients without malaria (OR = 0.80, p = 0.570). Therefore, presence of malaria parasitaemia was not associated with an increased risk of UTI in this study.

Among the patients who had parasitological-confirmed malaria infection, 10.6% had UTI (n/N), while 45.2% of the UTI cases occurred in patients who the clinicians managed as clinical malaria, though with a negative blood-slide for malaria parasites. No initial antibiotic was included in the admission plan for these patients. A clinical impression of UTI was made in 6 (1.6%) of all the assigned clinical diagnoses however only one of these patients had pyuria and a positive urine culture.

#### Etiologic agents cultured from urine samples

Of the 31 positive cultures, the majority of the isolates were Escherichia coli (20/31; 64.5%), followed by Staphylococcus aureus (4/31; 12.9%). Other isolates were Klebsiella pneumoniae (2/31; 6.5%), Pseudomonas spp (2/31; 6.5%), and Proteus spp. (1/31; 3.2%). Citrobacter spp (1/31; 3.2%) and Salmonella spp (1/31; 3.2%) were each isolated from inpatients. Four samples grew contaminants; one sample grew Lactobacillus spp, 2 samples had coagulase-negative staphylococci, and one had mixed growth.

#### Antimicrobial sensitivity

In vitro sensitivity testing showed high susceptibility to ciprofloxin, cefuroxime, ceftriaxone, gentamycin and nitrofurantoin (Table 5). However, resistance to several antibiotics was also observed; only a small fraction of isolates were sensitive to nalidixic acid (48.4%), amoxicillin clavulinate (25.8%), TMP-SMX (16.1%), amoxicillin (12.9%), and none were sensitive to ampicillin (0%).

**Table 5:** Overall antimicrobial sensitivity pattern.

ANTIBIOTIC	SENSITIVITY RESULTS		
	No. tested	SENSITIVE, N (%)	RESISTANT, N (%)
Ampicillin 10mcg*	26	0(0.0)	26(100)
**Amoxicillin 30mcg**	31	4(12.9)	27(87.1)
**TMP-SMX 25mcg**	31	5(16.1)	26(83.9)
**Amox-clav 20/10 mcg**	31	8(25.8)	23(74.2)
**Nalidixic acid 30mcg**	31	15(48.4)	16(51.6)
**Nitrofurantoin 300mcg**	31	25(83.3)	6(16.7)
**Gentamicin 10mcg**	31	25(83.3)	6(16.7)
**Ceftriaxone 30mcg**	31	30(96.8)	1(3.2)
Cefuroxime 30mcg*	7	7(100)	0(0)
**Ciprofloxacin 30mcg**	31	31(100)	0(0)

*Due to delays in acquiring cefuroxime and ampicillin antibiotic discs from suppliers, only 7 and 26 samples were tested against these drugs respectively.

#### Individual isolate sensitivity

Escherichia coli were isolated from 20 patients and all isolates were resistant to ampicillin and TMP SMX while at least 90% were also resistant to amoxicillin and amoxicillin clavulinate (Table 6).

**Table 6:** Individual isolate sensitivity to various drugs.

ANTIBIOTIC	SENSITIVITY PATTERN BY ISOLATE, N (n/N)						
	E. coli	S. aureus	Kleb.	Pseud.	Prot.	Salmon.	Citroba
	N = 20	N = 4	N = 2	N = 2	N = 1	N = 1	N = 1
**Ampicillin**	0/20	0/4	0/2	0/2	0/1	0/1	--
**Amoxicillin**	1/20	3/4	0/2	0/2	0/1	0/1	0/1
**TMP-SMX**	0/20	4/4	0/2	0/2	0/1	0/1	0/1
**Amoxclav**	2/20	4/4	0/2	1/2	1/1	0/1	0/1
**Nalidixic acid**	9/20	1/4	1/2	2/2	0/1	1/1	1/1
**Nitrofurant**	19/20	2/4	2/2	2/2	1/1	0/1	0/1
**Gentamicin**	18/20	3/4	2/2	2/2	1/1	0/1	0/1
**Cefuroxime**	5/20	1/4	--	--	1/1	--	--
**Ceftriaxone**	20/20	4/4	2/2	0/2	1/1	1/1	1/1
**Ciprofloxacin**	20/20	4/4	2/2	2/2	1/1	1/1	1/1

-- No isolates were tested in these cells.

## Discussion

Our study suggests Urinary Tract Infection caused by multi-drug resistant *Escherichia coli* and *Staphylococcus aureus* is common in febrile under 5year old children at Webuye Sub-county Hospital. Majority of UTIs are treated empirically, especially in developing countries where patients cannot afford consulting a doctor or having laboratory tests done. With increasing antimicrobial resistance, local susceptibility patterns of uropathogens should be available to guide appropriate antibiotics prescription.

The most common isolates from this study were *Escherichia coli* (64.5%) and *Staphylococcus aureus* (12.9%). This study corroborates findings from other studies showing the predominance of *Escherichia coli* (70–90%) in the etiology of community acquired UTI [23–26]. Other known non-*E*. *coli* gram negative organisms causing UTI in children include *Klebsiella*, *Proteus*, *Enterobacter* and *Pseudomonas* spp. Gram positive bacterial pathogens include *Enterococcus* and rarely *Staphylococcus aureus*. Our finding of a slightly lower occurrence of *Escherichia coli* and a higher than normal rate of non-*E coli* could be explained by probable high rate of exposure to antibiotics at the community level. A lower rate of occurrence of *Escherichia coli* has been shown in children on prophylactic antibiotics (58%) and those with a history of UTI (74%) [27] signifying the effect of prior exposure to antibiotics on etiologic patterns. In a review of the clinical and laboratory characteristics of infants and children aged one week to 16 years with UTI caused by *Escherichia coli* compared with other pathogens, *non-E*. *coli* UTI was associated with urinary tract anomalies, younger age and previous antibiotic use [28].

Contrary to our findings, *Staphylococcus aureus* is not thought to be a common pathogen in UTI. However other studies in children in developing countries have shown an increasing prevalence of *Staphylococcus aureus* isolated in children with UTI [29–31]. The predominance of this organism could imply changing pattern of uropathogens. Other possibilities include hematogenous infection and possible contamination from the skin. Nonetheless, since this study had only four isolates, the finding requires further investigation.

Worryingly, we found high rates of resistance to first-line antibiotics. *Escherichia coli*, the most common isolate, was found to be resistant to ampicillin, amoxicillin, TMP-SMX, amoxicillin-clavulanate and nalidixic acid. These are commonly prescribed antibiotics at peripheral outpatient health facilities and are also readily obtained over the counter in drug shops, pharmacies and chemist. A recent study within the region has reported a resistance rate of up to 98.4% of *Escherichia coli* from urine samples among febrile children [32]. *Escherichia coli* maintained high sensitivities to gentamycin (90%), nitrofurantoin (95%), cefuroxime (100%), ceftriaxone (100%), and ciprofloxacin (100%) in this study.

The high rate of resistance of *Escherichia coli* to commonly used antibiotics and the emergence of *Staphylococcus aureus* as a significant causative agent could possibly indicate a problem at the community or facility level either due to wrong prescription, over-prescription, or poor adherence. Two (5%) of the patients excluded in this study for having used antibiotics still had pyuria and a lower than standard growth of bacteriuria on culture. These were managed as UTI but excluded from our final analysis. This highlights an urgent need for antibiotic stewardship and continuous local epidemiologic surveillance to monitor antimicrobial resistance patterns.

The overall prevalence of UTI in our study population was 11.9%. Our findings are largely in line with other studies, however there is substantial regional variation in the prevalence of UTI in the developing world depending on setting, ranging from 13.7% to 19.3% [23,33,34] compared to reported prevalence of 3.3%-9% in developed countries [24,35,36]

A study done in Nairobi, Kenya found UTI prevalent in 13.3% of children with malaria [15]. Despite the similarity in prevalence estimates, there were some methodological differences between our findings and the study by Okwara and colleagues. The study used urine bag samples in children younger than 2years of age. Studies have shown culture of such urine specimens has an unacceptably high false positive rate and are valid only when they yield negative results [37, 38]. Kebira et al in 2009 using the criteria of significant bacteriuria and pyuria to define UTI found a higher prevalence of 16% in children aged 1-4years attending both outpatient and inpatient services at Thika Sub-county Hospital, Kenya [39], although they also used urine bags for children less than 3 years. The prevalence from our study was lower than the 20.3% [25] and 29.3% [40] recently reported in East Africa.

Awareness of the prevalence of UTI in various demographic sub-groups of children enables the clinician to estimate the probability of infection in the patient. We found a pooled prevalence of 10.1% in children below 2years while those older than 2 years had 13.8%. Studies have shown that urine requires at least 4hours of incubation in the urinary bladder to achieve significant growth on culture and for dipstick to be positive for Nitrites [41]. Since dipstick was used as screening test in this study, we may have excluded some patients with UTI especially in the younger category who are not toilet trained to hold urine in the urinary bladder for longer hours. Furthermore, studies show that approximately 3% of UTI could be missed when combined dipstick test for LE and N are used as screening tool [42].

Fever has been reported as the commonest symptom in under- five children with UTI and a temperature increase above 38°C associated with the infection [16, 32, 35, 43]. We found this similarity with 74.2% of children with UTI presenting with a temperature of 38°C and above. We did not find a significant difference in prevalence of UTI between male and female children, but this may be because the majority of the male children were uncircumcised and that our sample size was small. We found a significantly higher prevalence of UTI in admitted patients as compared to outpatients. It is probable that children who were ill enough to be hospitalized had multiple infections, including UTI.

This study further corroborates findings from other studies showing that UTI frequently complicates other childhood febrile illnesses, especially malaria [25, 44]. We found that 10.6% of UTI cases occurred in children with a smear positive for malaria, which was similar to estimates in other studies [25, 29, 32]. Findings from a study in South Africa showed that 86% of UTI cases occurred in association with other serious clinical conditions [45, 46].

It is not possible to accurately diagnose UTI using clinical criteria alone in under 5year old children, since the presentation is non-specific. Studies have developed clinical prediction rules [16] for young infants with possible UTI but their sensitivity and specificity is low. In our study, a clinical impression of UTI was made in only 6 patients and overall the clinicians were correct in less than 5% of the total UTI cases. Furthermore, 45.2% of the UTI cases occurred in a group that clinicians entertained an impression of clinical malaria, despite a negative blood-slide for malaria parasites. UTI diagnosis would have been missed or its treatment delayed had no effort been made to investigate.

Limitations to this study included reliance on reported history of antibiotic use from parents or guardians. This may have been negatively affected by social desirability factors ultimately affecting the yield on urine cultures. We also used dipstick and microscopy as screening tests which may have missed some UTI cases. Urine requires at least 4hours of incubation in the urinary bladder to achieve significant growth on culture and for dipstick to be positive for Nitrites. Since dipstick was used as screening test in this study, we may have excluded some patients with UTI especially in the younger category who are not toilet trained to hold urine in the urinary bladder for longer hours. There was also delay in acquiring two antimicrobial discs from suppliers affecting the total number of tests for these two. Urine sample collection in under-two year old girls was a challenge and may have contributed to the four contaminants.

## Conclusion

Our study demonstrates a significant burden of UTI in young children which often co-occurs with other infections. Since clinical presentation alone is not sufficient to make an accurate impression of UTI in young children clinicians must exercise a high index of suspicion and make deliberate efforts to investigate for UTI in children especially among inpatients and those in whom an antibiotic is considered. The choice of antimicrobial therapy should be based on available local sensitivities or the patient’s urine culture isolate. When treatment is started empirically the antibiotic chosen should provide adequate coverage for *Escherichia coli*, the most common bacterial cause of UTI.

## Supporting information

S1 File Minimum data for the study, excel spreadsheet. (XLSX)

S2 File Minimum data, STATA summaries. (TXT)
